# Reversible perturbations of gene regulation after genome editing in *Drosophila* cells

**DOI:** 10.1371/journal.pone.0180135

**Published:** 2017-06-28

**Authors:** Stefan Kunzelmann, Klaus Förstemann

**Affiliations:** Department of Biochemistry, Gene Center, Ludwig-Maximilians-Universität München, München, Germany; Lewis Katz School of Medicine at Temple University, UNITED STATES

## Abstract

The prokaryotic phage defense CRISPR/*cas*-system has developed into a versatile toolbox for genome engineering and genetic studies in many organisms. While many efforts were spent on analyzing the consequences of off-target effects, only few studies addressed side-effects that occur due to the targeted manipulation of the genome. Here, we show that the CRISPR/*cas9*-mediated integration of an epitope tag in combination with a selection cassette can trigger an siRNA-mediated, epigenetic genome surveillance pathway in *Drosophila melanogaster* cells. After homology-directed insertion of the sequence coding for the epitope tag and the selection marker, a moderate level of siRNAs covering the inserted sequence and extending into the targeted locus was detected. This response affected protein levels less than two-fold and it persisted even after single cell cloning. However, removal of the selection cassette abolished the siRNA generation, demonstrating that this response is reversible. Consistently, marker-free genome engineering did not trigger the same surveillance mechanism. These two observations indicate that the selection cassette we employed induces an aberrant transcriptional arrangement and ultimately sets off the siRNA production. There have been prior concerns about undesirable effects induced by selection markers, but fortunately we were able to show that at least one of the epigenetic changes reverts as the marker gene is excised. Although the effects observed were rather weak (less than twofold de-repression upon *ago2* or *dcr-2* knock-down), we recommend that when selection markers are used during genome editing, a strategy for their subsequent removal should always be included.

## Introduction

The CRISPR/*cas*-system has become an indispensable method to manipulate genomes with comparably little effort and few side effects. It allows for the generation of mutant chromosomal loci as well as epitope tag knock-ins. Concerns were raised about possible off-target effects and their consequences on experimental results [1, 2]. In contrast, we know little about how organisms deal with the on-target manipulation once it is in place. Do the cells “recognize” inserted sequences and respond to these foreign elements?

The artificial manipulations bear similarities with transposable elements (TEs), which are naturally occurring insertion events that threaten genomic stability. TEs code for enzymes that mobilize and re-insert them in new genomic locations. Cells have developed several defense strategies to suppress transposition [3]. For example, in somatic cells of *Drosophila melanogaster*, the RNA interference (RNAi) pathway is responsible for the posttranscriptional silencing of TEs. Double-stranded RNA (dsRNA) precursors, which derive e.g. from transcription events of structured loci or convergent transcription, are processed by the endonuclease Dicer-2 (Dcr-2) into 21 nt long siRNAs [4–10]. These small RNAs are then loaded into Argonaute 2 (Ago2) and direct this RNA-induced silencing complex (RISC) to mRNAs bearing perfect complementarity. Ago2 cuts the mRNA, which is then degraded [11].

To address the question how the cells deal with genetic manipulations using the CRISPR/*cas9*-mediated genome editing approach, we used *Drosophila melanogaster* S2 cells as a model system and the epitope tag knock-in protocol of our lab that is based on homologous recombination (HR) donors generated by PCR with short homology arms [12, 13]. We were able to show that *bona fide* siRNAs are generated upon insertion of homologous recombination donors with selection cassettes. These siRNAs target predominantly the inserted sequence but also spread to adjacent transcribed region. Importantly, this siRNA response disappears upon removal of the selection cassette and marker free tagging circumvents siRNA production altogether. Despite the downside of triggering ectopic siRNA production, selection cassettes greatly facilitate the enrichment of cells carrying the desired genome modification. Fortunately, the undesired RNAi response can be fully reversed by excision of the selection cassette. It is thus possible to benefit from the use of selection markers, but nonetheless avoid at least some of the associated liabilities.

## Materials& methods

### Cell culture, genome editing and cloning

*Drosophila melanogaster* Schneider cells (laboratory stock) were cultured in Schneider’s medium (Bio&Sell, Germany) containing 10% fetal bovine serum (Biochrom, Germany). CRISPR/*cas9*-mediated genome editing with 60 nt homology PCR products was performed as previously described [12, 13]. Primer sequences are provided in S1 Table. For single cell cloning, cells were seeded at 10,000 / ml in 25% conditioned medium and plated in serial dilutions (1:2). Single cell colonies were picked and cultured for further analyses. Please note that we provide a detailed experimental protocol for the generation of genome-edited S2-cells in our 2016 G3 (Bethesda) publication [13].

### Reporter assay

Cells were treated for 7 days with previously with validated *in vitro* transcribed dsRNA to induce gene specific knock-downs [4, 14, 15]. For quantification of GFP fluorescence, 100 μl of cells were resuspended in 500 μl of FACS Flow, then measured in a Becton Dickinson FACSCalibur flow cytometer as previously published [12]. The data was analyzed using Flowing Software version 2.5.1 (http://www.flowingsoftware.com), subsequent calculations were done in Microsoft Excel.

### Library generation, deep sequencing and data analysis

sRNA libraries were generated essentially as previously described [16]. However, the ZR small RNA PAGE Recovery Kit (Zymo Research, USA) was used after PAGE-purification of the small RNAs. Deep sequencing was performed on an Illumina HiSeq instrument by LAFUGA (Gene Center, LMU Munich, Germany) and the sequences were analyzed using custom Galaxy, bowtie, perl and R scripts (available upon request). The original sequencing data has been deposited at the European Nucleotide Archive with the accession number PRJEB20499.

## Results and discussion

### Functional siRNAs target integrated epitope-tag cassettes

**Fig 1:**
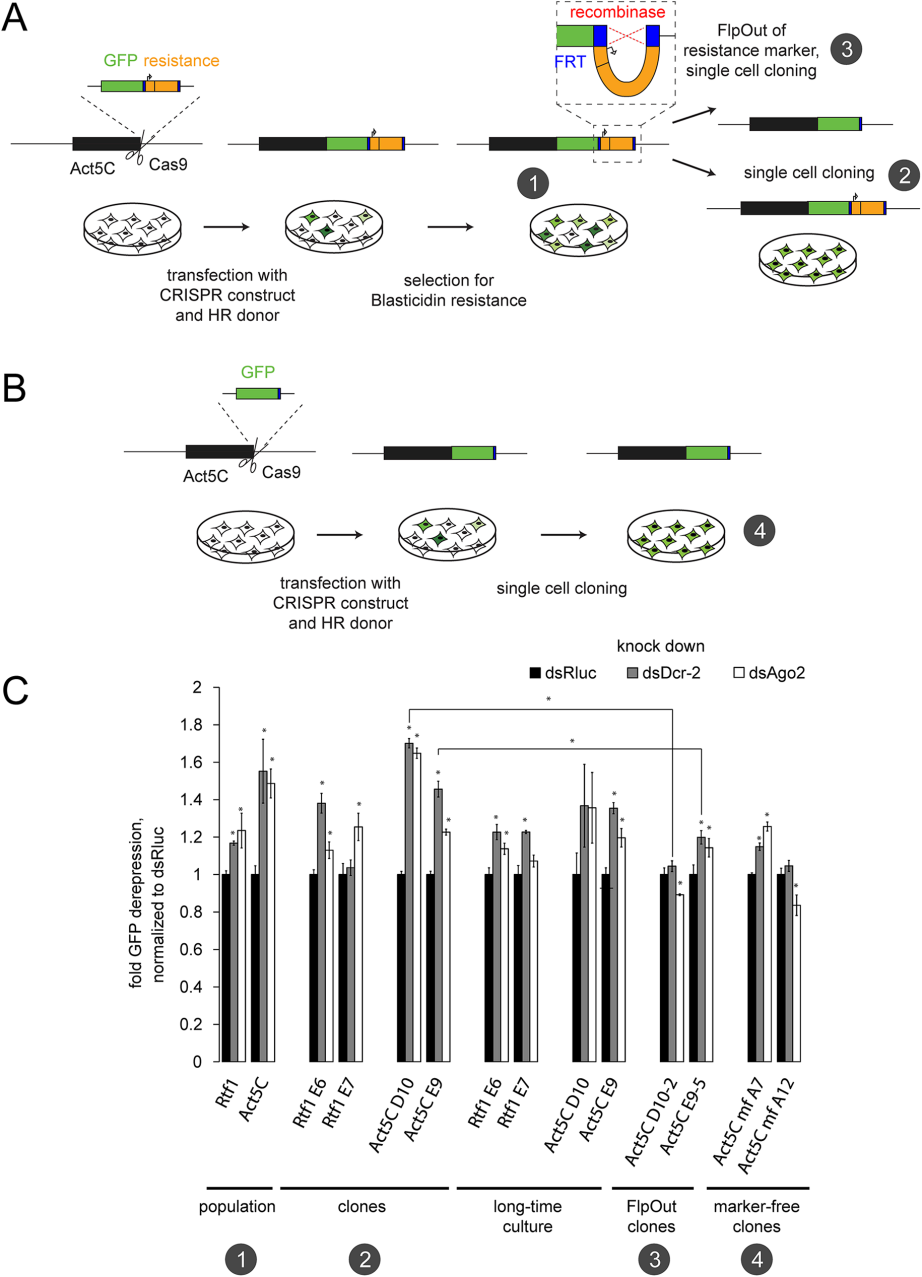
GFP-based reporter assay can detect siRNA mediated repression after genome engineering. (A) PCR-based tagging workflow using CRISPR/Cas9 in *Drosophila* Schneider cells. After introducing a DSB at the *act5C* locus by the Cas9 enzyme, the HR donor (consisting of homology regions, the GFP coding sequence and the resistance cassette) integrates and GFP-positive cells can be eriched by drug selection (number 1) and cloned (number 2). The recombinase mediates the FlpOut of the resistance cassette and subsequent single cell cloning results in FlpOut clones (number 3). (B) Marker-free tagging of the *act5C* locus with GFP. Similar to (A), the *act5C* locus can be tagged without an selection marker. Single cell cloning resulted in homogeneous cell lines (number 4). (C) GFP-based reporter assay detecting the presence of functional siRNAs in several cell lines. Knockdown of Dcr-2 and Ago2 as key players of the RNAi pathway leads to derepression of the GFP fluoresence in the twoAct5C-GFP and Rtf1-GFP cell lines. Fluorescence levels (FL1 channel) were normalized to control knockdown (Rluc). Error bars represent standard deviation (n = 3). Significant differences were calculated though an unpaired t-test (unequal variance) on the data (* p < 0.05).

We previously developed a CRISPR/*cas9*-mediated genome editing workflow for *Drosophila* cell culture to introduce epitope tags adjacent to the coding sequences of genes at their chromosomal loci (Fig 1A and 1B). After enrichment of positive cells by antibiotic selection, the resistance marker can be removed via the Flp/FRT system [12, 13]. In order to study the potential of modified loci to trigger siRNA generation, we introduced a C-terminal GFP-tag at the *act5C* and *rtf1* loci in S2 cells. If these foreign sequences are targeted by siRNAs, then the GFP-fusion proteins should be de-repressed upon inactivation of the RNAi pathway. Although it may seem counter-intuitive at first, depletion of siRNA biogenesis factors is possible via RNAi itself as first demonstrated in *C*. *elegans* [17, 18]. We thus monitored GFP expression of the RNAi-treated, genome-edited cells with flow cytometry. Knockdown of the siRNA biogenesis enzyme Dcr-2 as well as the effector protein Ago2 with previously validated RNAi triggers [4, 14, 15] resulted in derepression of the Act5C-GFP and Rtf1-GFP fusion proteins. This effect was less than two-fold, already visible in the cell population after one split into selective medium and remained after clonal selection. Even after prolonged cultivation (12 weeks) of these cell lines without selection pressure, the effect did not vanish (Fig 1C, stages 1 and 2). This argues for a stable situation that is not transiently triggered by the induced DNA double-strand break.

**Fig 2:**
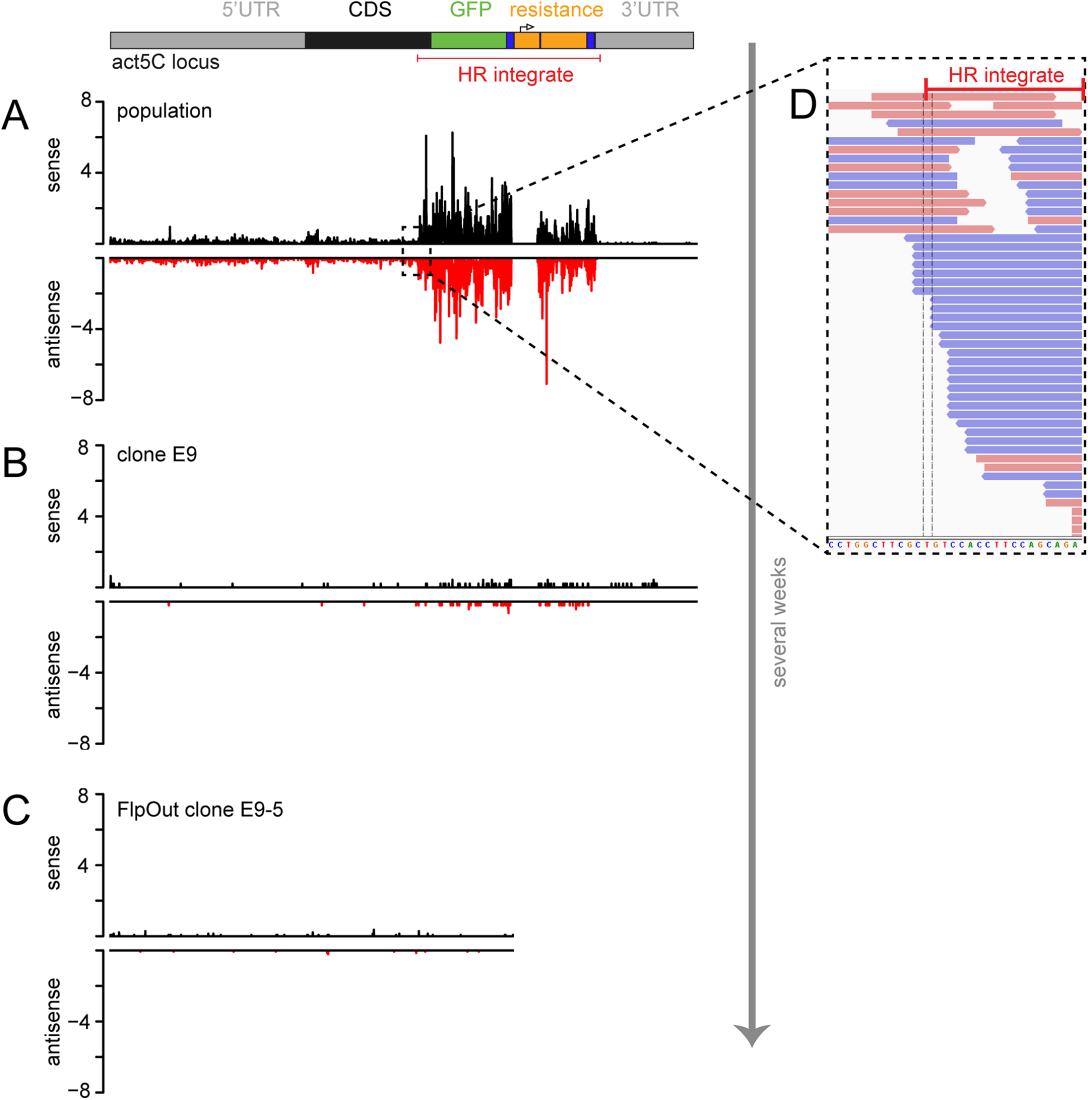
Profiling of siRNAs after genome editing by deep sequencing at the *act5C* locus. The siRNA distribution along the modified *act5C* locus was determined bat single nucleotide resolution and normalized to the number of genome-matching reads in each library. The graphs depict the sense (black) and antisense (red) matching reads as reads per million of genome matching 19–25 nt reads in the respective library. Shown are the sequencing traces for the initial drug-selected population (A), the single cell clone E9 (B) and the respective FlpOut clone E9-5 (C) as representive examples. The functional regions of the locus (drawn to scale) are depicted at the top; the HR donor is annotated in red. Reads derived from the *copia* promotor sequence were removed prior to mapping the remaining reads on the construct. Thus, the corresponding region is “masked”. The box (D) shows the magnification of the transition between the endogenous sequence and the HR integrate (annotated red bar). Junction-spanning siRNA reads in sense (red) and antisense (blue) orientation can be detected.

**Fig 3:**
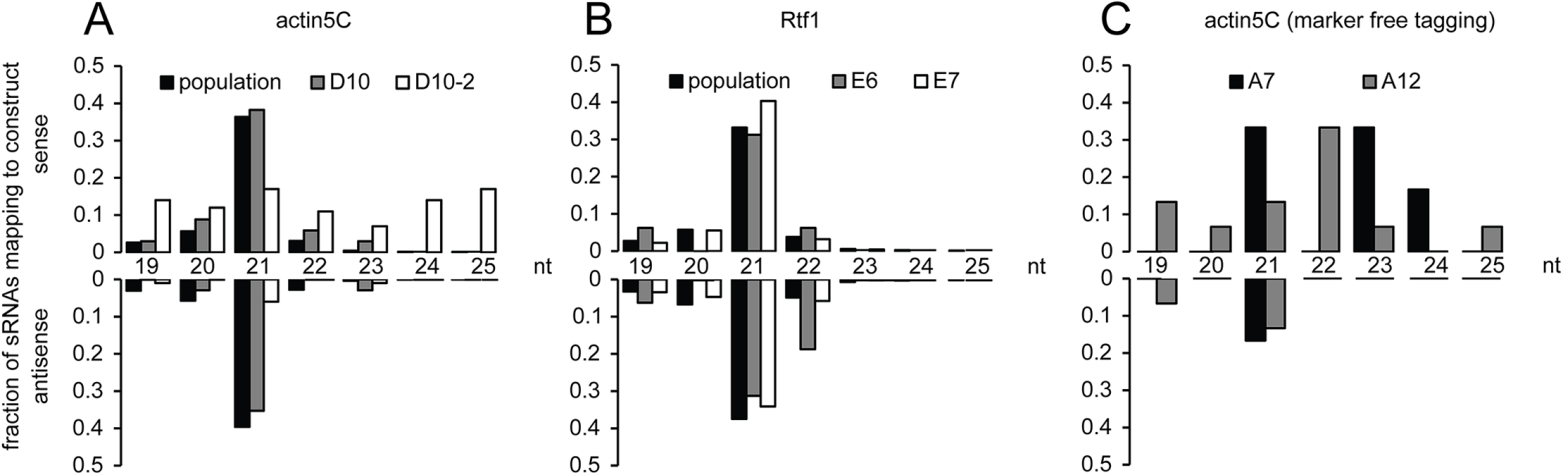
**Read length distribution of *act5C* (A, C) and *rtf1* (B) locus matching reads in sense and antisense orientation of representative cell lines.** Data is presented as fraction of total siRNAs mapping to the construct. (Actin5C D10 = clone, D10-2 = FlpOut clone; Rtf1E6 and E7 = clones; Actin5C A7 and A12 = marker-free tagged clones).

We sequenced the small RNA profile of the genome-engineered cell lines and mapped the reads back to the modified loci. This provided direct evidence for the presence of small RNAs in sense and antisense orientation targeting the *act5C* locus (Fig 2) or the *rtf1* locus in cells of the drug-selected population as well as single cell clones. We first examined the size distribution of the reads that were mapped to the locus. They showed a clear peak of 21 nt long reads in sense and antisense orientation (Fig 3). Together with their Dcr-2 and Ago2 dependent activity, this argues for *bona fide* siRNAs.

**Fig 4:**
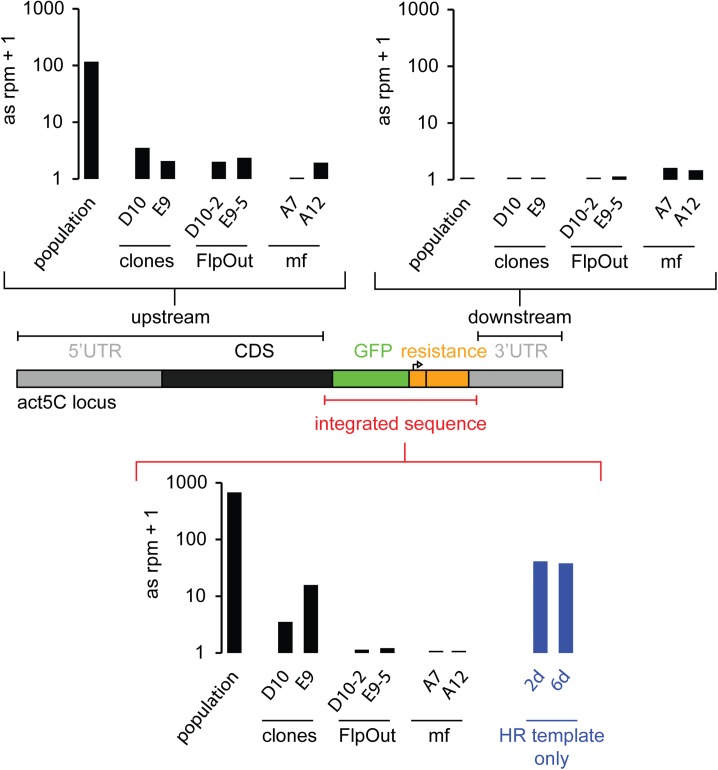
Quantification of the siRNA strength at the *act5C* locus for different cell lines. Sequenced siRNAs were mapped to the modified loci and antisense reads (only) mapping either to the upstream or downstrem region of the integrated sequence or the HR donor were summed up and normalized to genome matching reads and length of the sequence region. (mf = marker-free tagged cell lines, FlpOut = FlpOut cell lines).

**Fig 5:**
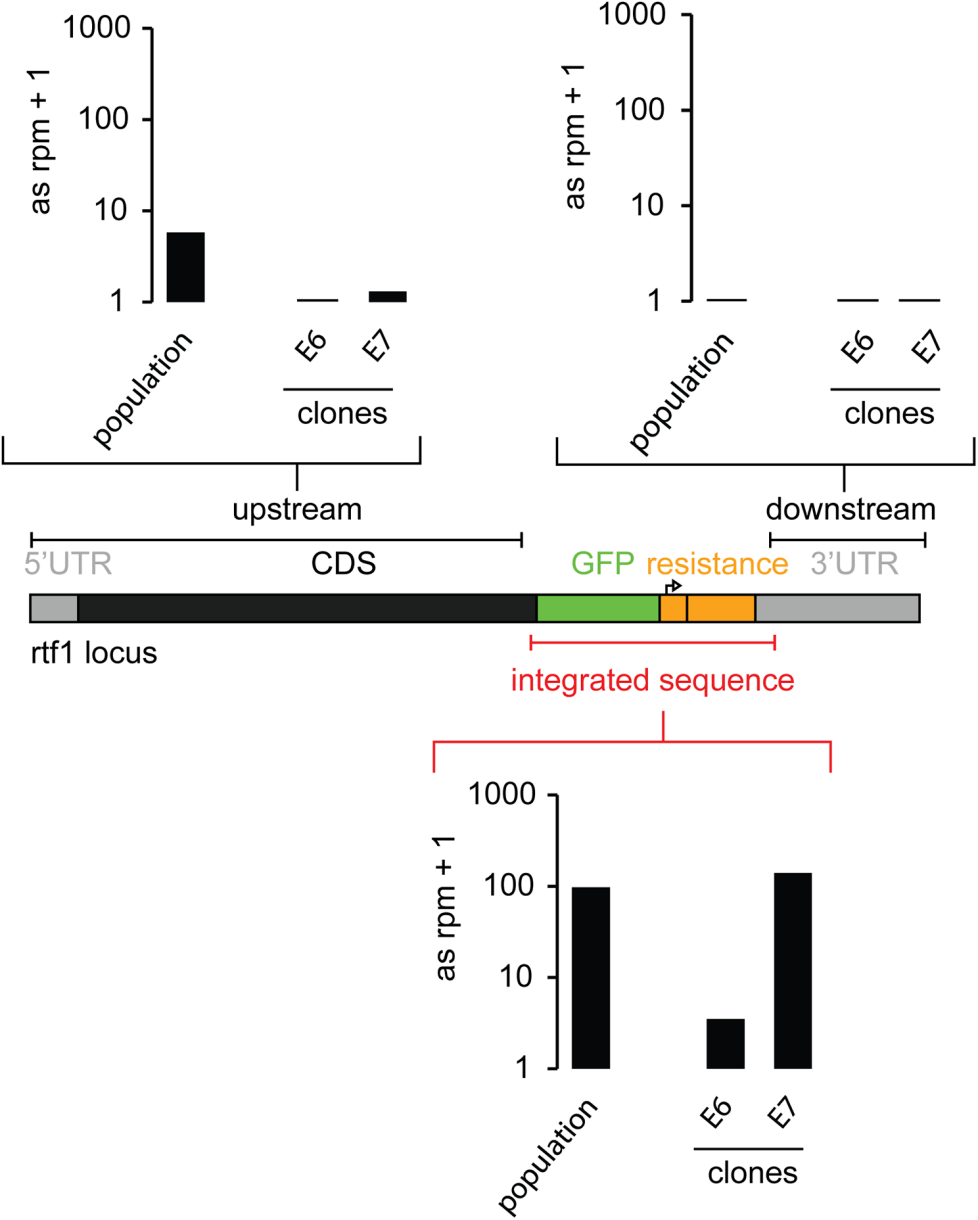
Quantification of the siRNA strength at the *rtf1* locus for different cell lines. Sequenced siRNAs were mapped to the modified loci and antisense reads mapping either to the upstream or downstrem region of the integrated sequence or the HR donor were summed up and normalized to genome matching reads and length of the sequence region.

Since sense matching reads can also be mRNA degradation products, we quantified the strength of the siRNA response by summing up only antisense reads mapping to either the HR integrate (= the HR donor after integration), the upstream sequence or the downstream sequence of this locus (Figs 4 and 5). The majority of siRNAs derived from the HR integrate, but reads also mapped upstream of the integration site. In particular, we found reads in antisense orientation that span the junction between the HR integrate and the *act5C* host gene (Fig 2D). This suggested that the dsRNA precursor of the siRNAs extends beyond the inserted sequence and excludes off-target integration events as being the predominant source of those siRNAs. The strength of the siRNA response decreased after clonal selection compared with the initial drug-selected population after genome editing. Nevertheless, the measurement of GFP fusion protein levels after Dcr-2 and Ago2 knock-down proved the potential of the remaining siRNAs to act as repressors (Fig 1C). It depends on the particular situation if these small changes in expression levels can interfere with experimental results and introduce biases to studies. Nevertheless, they may be an indicator that further epigenetic changes may have occurred at the modified locus.

### Excision of the selection cassettes removes the siRNA trigger

We then tested whether specific parts of the introduced sequence were responsible for triggering the siRNA generation. To this end, we used Flp recombinase to remove the FRT-flanked selection cassette, which consists of the *copia* promotor and the Blasticidin resistance gene (see Fig 1A, stage 3). The “Flp-out” of the selection cassette resulted in loss of small RNAs repressing the fusion protein, observed both in the GFP-based expression assay and by small RNA sequencing (Fig 1C, Fig 4). The remaining small RNAs were predominantly sense oriented and did not show an accumulation of 21 nt long reads; most likely, they represent mRNA degradation products (Fig 3A, clone D10-2).

To further validate the hypothesis that the resistance cassette is the trigger for siRNA biogenesis, we generated GFP-tagged *act5C* clones after marker-free genome editing (Fig 1B). We employed the same template plasmid but a different homology-containing antisense primer to generate HR donor PCR products that only contained the GFP coding sequence and the homology arms. After transfecting our Cas9-expressing cell line with the sgRNA expression construct and the HR donor, we established Act5C-GFP positive cell lines by single cell cloning and visual screening. From the initial 93 hand-picked clones, two lines had the desired *act5C*-GFP modification. The Act5C-GFP fusion protein neither showed Dcr-2 and Ago2 dependent repression (Fig 1C, stage 4), nor did we detect any corresponding siRNA reads by small RNA sequencing (Fig 4). Thus, it is not the tagging process *per se* that is responsible for the siRNA response, but rather the selection cassette comprising a promoter and resistance gene. The *copia* promoter, which drives expression of the Blasticidin resistance in our cassette, has sequence identity with an endogenous transposable element that is constitutively targeted by siRNAs (note that we excluded this region in our siRNA sequencing analysis). It is conceivable that these siRNAs serve to nucleate a response that then spreads into the surrounding sequence analogous to siRNA-directed heterochromatin formation in fission yeast [19, 20]. However, since the cassette excision completely reverts the siRNA generation, we favor the hypothesis that a low-level of antisense transcription activity of the *copia* promoter causes convergent transcription with the host gene and thus the generation of dsRNA at the site of integration. Whatever the precise molecular mechanism may be, we recommend implementing strategies for removal of selection cassettes where possible.

### Integration of the HR donor is a prerequisite for the generation of siRNAs

In higher eukaryotes, defense mechanisms target linear dsDNA in a context of DNA virus infection [21, 22] and RNA polymerase III can serve as a sensor for cytoplasmic DNA [23]. We thus tested if the introduction of a linear PCR product, the HR donor used for GFP-tagging at the *act5C* locus, without a corresponding Cas9-mediated cut in the DNA is sufficient to trigger the generation of siRNAs. Small RNAs were sequenced two and six days after transfection and the sense and antisense reads mapping to the PCR product were quantified. In contrast to the robust response we detected at a comparable time point for the productively genome modified Act5C-GFP cell population (~680 reads per million genome matching sequences, rpm), the response was approximately 15-fold weaker (40 rpm) when the HR-stimulating site-specific DNA cut was omitted (Fig 4). Together with our observation that siRNAs repress the targeted locus even after prolonged culture, when all non-replicated sequences have been lost, this argues against a major contribution of episomal DNA to the siRNA pool.

## Conclusions

We describe the induction of an siRNA response after genome editing in cultured *Drosophila* cells. This response is elicited by the selection cassette, which serves to enrich for cells with the desired modification. Fortunately, removal of the FRT-flanked cassette with FLP recombinase abolished this response. The same result was obtained when genome editing was performed without selectable markers. Our measurements of GFP-fusion protein levels argue that the quantitative extent of siRNA-mediated repression is less than two-fold. This may compare to the effect of a heterozygous, recessive loss-of-function mutation. To insert a C-terminal tag, the marker cassette truncates the endogenous 3’-UTR; for N-terminal tags a surrogate promoter for expression of the fusion protein must be provided. It can be expected that gene expression is more heavily affected by the ensuing disturbance of transcriptional or post-transcriptional regulation than by the concomitantly induced siRNAs. There are thus several reasons why removal of the selection cassette is a “best practice” to follow if the least invasive genome modification is the aim. Our finding that in cultured cells even the epigenetic phenomenon of RNA interference can be reversed is an encouraging observation: It may be possible to benefit from the advantages of marker selection without inducing irreversible changes in gene expression. Nonetheless, perturbations of the targeted protein’s stability and/or functionality by the appended epitope tag remain a concern that should be experimentally addressed.

## Supporting information

S1 Table Oligonucleotide sequences used for this study. (XLSX)
